# Identification and characterization of Csa-miR159s and their expression patterns under different abiotic stresses in cucumber (*Cucumis sativus* L.)

**DOI:** 10.3389/fpls.2025.1518406

**Published:** 2025-04-24

**Authors:** Zhenxiang Zhao, Wenhong Ao, Weirong Luo, Yaoguang Sun, Vijay Yadav Tokala, Junjun Liu, Shenshen Zhi, Yongdong Sun

**Affiliations:** ^1^ School of Horticulture and Landscape Architecture, Henan Institute of Science and Technology, Xinxiang, China; ^2^ Henan Province Engineering Research Center of Horticultural Plant Resource Utilization and Germplasm Enhancement, Henan Institute of Science and Technology, Xinxiang, China; ^3^ Horticulture Research and Extension, The Postharvest Education Foundation, La Pine, OR, United States

**Keywords:** cucumber (*Cucumis sativus.* L), Csa-miR159s, fruit expansion, abiotic stress, phytohormones

## Abstract

The miR159 gene family plays an essential role in plant growth and development, and stress response. Nevertheless, there are no reports defining its specific function in cucumber fruit expansion and response to abiotic stresses. In this study, we retrieved six Csa-miR159 sequences from the EnsemblPlants database, which were located on chromosome 1, chromosome 3, and chromosome 5 of cucumber, respectively. Phylogenetic analysis showed that Csa-miR159c/d/e/f belonged to one branch and Csa-miR159a/b to another. *C*is-acting regulatory elements (CREs) including light response elements, phytohormone response elements, stress response elements, regulatory elements associated with plant growth and development were distributed unevenly in the promoter regions of Csa-miR159s, which indicated that Csa-miR159s might mediate the stress response, and growth and development. Moreover, it was determined that *CsMYBs* were the target genes of Csa-miR159s through psRNA-Target prediction and qRT-PCR analysis. Further findings suggested that Csa-miR159b might negatively regulate cucumber fruit expansion by targeting *Cs1RMYB9*, *Cs1RMYB31*, *Cs2RMYB37* and *Cs2RMYB64*. Similarly, Csa-miR159d might negatively regulate cucumber fruit expansion by targeting *Cs2RMYB27* and *Cs2RMYB32*. In addition, the differential expression of Csa-miR159s suggested their potential response to abiotic stresses and plant phytohormones. This study would provide valuable information on the molecular characterization of Csa-miR159s and establish a foundation for further research on the mechanisms of Csa-miR159s in regulating fruit expansion and stress response.

## 1 Introduction

Cucumber (*Cucumis sativus* L.) is a widely cultivated vegetable worldwide, with China being the largest producer and consumer. In 2023, China cultivated approximately 1.37 million hectares of cucumber, with a total yield of 80.21 million tons. This accounted for 60.40% of the global cucumber cultivation area and 82.01% of the total production (Food and Agriculture Organization of the United Nations, 2023). However, unfavorable environmental conditions such as inappropriate temperature, weak light, drought and salt stresses, have a significant impact on cucumber fruit expansion, leading to the decreased yield and quality. Therefore, it is important to understand the molecular mechanism of cucumber fruit expansion in order to improve yield and stress tolerance in cucumber.

MicroRNA (miRNA) is a class of highly conserved endogenous non-coding small RNA, regulating the expression of its target genes at both the levels of transcription and post-transcription by directly cleaving or inhibiting the translation of target mRNA (Jones-Rhoades et al., 2006). It plays crucial roles in various physiological and metabolic processes, such as plant growth and development, and stress response (Jeong and Green, 2013; Liu et al., 2007; Zhang et al., 2019). miR159 has been extensively studied in plants (Montes et al., 2014) and the studies reveal how it influences plant growth and development by targeting *MYB* family genes (Dubos et al., 2010). For instance, miR159-*GAMYB* pathway has been widely implicated in plant growth, stress response, and phytohormone signaling in various species such as *Arabidopsis* (Allen et al., 2010, 2007; Alonso-Peral et al., 2010), tomato (Zhang et al., 2020) and rice (Zhao et al., 2017). In Gloxinia (*Sinningia* sp*eciosa*), expression patterns of miR159 and *GAMYB* were negatively correlated during flower development (Li et al., 2013). In addition, some studies have demonstrated the important role of the miR159-*GAMYB* in fruit development. For instance, in tomato, overexpression of Sl-MIR159 led to the down-regulation of *SlGAMYB*, thereby inducing parthenocarpy and early fruit ripening (da Silva et al., 2017). Similarly, Sly-miR159-*SlGAMYB2* was also found to control fruit growth, as the inhibition of Sly-miR159 and overexpression of *SlGAMYB2* resulted in the larger fruit, while the loss of function of *SlGAMYB2* led to the smaller fruit (Zhao et al., 2022). In the case of grape, exogenous application of gibberellin (GA) promoted parthenocarpy, accompanied by the up-regulation of Vvi-miR159c and the down-regulation of *VvGAMYB* (Wang et al., 2018).

The miR159-*GAMYB* pathway is known to play a crucial role in the response to drought and salt stresses. Studies have shown that miR159 was induced by drought stress in plants such as *Arabidopsis* (Reyes and Chua, 2007), maize (Wei et al., 2009), wheat (Akdogan et al., 2016), barley (Hackenberg et al., 2015) and poplar (Fu et al., 2023). However, in potato, the expression level of miR159 decreased under drought treatment, while the expression level of *GAMYB*-like homologues increased (Pieczynski et al., 2013). *SlMYB33*, the target gene of Sly-miR159, was associated with the accumulation of proline and putrescine, which enhanced plant tolerance to drought stress (López-Galiano et al., 2019). Furthermore, it has been reported that miR159 can be induced by salt stress in *Arabidopsis* (Liu et al., 2008) and soybean (Li et al., 2023). Additionally, miR159-*GAMYB* plays a crucial role in some plant phytohormone signaling pathways, such as abscisic acid (ABA) (Reyes and Chua, 2007) and GA (Wang et al., 2017). For instance, in ‘Zuijinxiang’ grape, the expression level of VvimiR159 increased after GA treatment, while the expression level of *VvGAMYB* significantly decreased (Wang et al., 2018). In ‘Rosario Bianco’ grape, the expression of miR159 was up-regulated in the pulp after GA treatment, whereas the expression of miR159a/c was down-regulated in the pulp and pericarp (Han et al., 2014). Overall, the miR159-*GAMYB* pathway plays a role in response to abiotic stresses and plant phytohormones. In our previous study, differential expression of Csa-miR159b was observed between the ovary and expanded fruit using small RNA sequencing, which suggested that Csa-miR159b was involved in cucumber fruit expansion (Sun et al., 2019). However, there was a scarcity of studies on the functions of Csa-miR159s in relation to cucumber fruit expansion and stress response.

This study aims to characterize Csa-miR159s in cucumber and to investigate their roles in fruit expansion and stress response. In the present study, multiple sequence alignment, chromosomal location, secondary structure, phylogenetic relationship, *cis*-regulatory elements (CREs), and the target genes of Csa-miR159s were studied in detail. Additionally, expression profiles of Csa-miR159s were analyzed in the ovary and expanded fruit, and in response to different stresses and plant phytohormones. Our findings will provide valuable information for further functional analysis of Csa-miR159s in cucumber, and also provide references for improving cucumber yield and resilience.

## 2 Materials and methods

### 2.1 Identification of Csa-miR159s

A search for miR159 family members in cucumber was conducted using EnsemblPlants database (http://plants.ensembl.org/). The mature sequences of miR159s from various crop species (zucchini, watermelon, pumpkin, cucumber, melon, tomato, rice and *Arabidopsis*) were obtained from the PmiREN database (Guo et al., 2020). Multiple sequence alignments of Csa-miR159s were performed using ClustalW software (Thompson et al., 1994), and were used to generate a sequence logo diagram through the online website (https://weblogo.berkeley.edu/). TBtools software (Chen et al., 2023) was employed to visualize the distribution of Csa-miR159s on cucumber chromosomes. The RNA secondary structure of pre-MIR159s was predicted using the RNAstructure web server (http://rna.urmc.rochester.edu/RNAstructureWebServers/Predictl/Predictl.html). Mature sequences of miR159s were submitted to MEGA v5.1 software (Kumar et al., 2018) to construct phylogenetic relationships using the neighbor-joining (NJ) method with 1000 bootstrap replicates to assess branch confidence. The 2000 bp promoter sequences upstream from the initiation codon of Csa-MIR159s were extracted from EnsemblPlants database. The putative *cis*-regulatory elements (CREs) were identified and analyzed using the PlantCARE tool (http://bioinformatics.psb.ugent.be/webtools/plantcare/html/) (Lescot et al., 2002).

### 2.2 Prediction of target genes

psRNA-Target uses sequence complementarity and energy-based scoring to predict miRNA-target interactions. A score threshold of ≤5.0 was chosen based on established standards for psRNA-Target to ensure high confidence in predicted interactions. To predict the potential target relationships of Csa-miR159s and *CsMYBs*, their gene sequences were submitted to the psRNA-Target online website (https://www.zhaolab.org/psRNATarget/) (Liu et al., 2015), and target genes with a score ≤5.0 was selected and submitted to Cucurbit Genomics Database (CuGenDB) for further analysis.

### 2.3 Plant growth conditions and stress treatments

Seeds of cucumber (cv. Jinyou No. 1) were soaked in water at a temperature of 55°C for 15 min and then incubated at 28°C for 2 days to germinate. The germinated seeds were cultivated in a pot filled with a medium consisting of peat soil, perlite, and vermiculite in a 2:1:1 ratio, and placed in a climate-controlled chamber at a temperature of 28°C with a light period of 16 h and a dark period of 8 h. Cucumber seedlings at the three-leaf stage were transferred to the plastic greenhouse for continuous growth. Samples from ovary (on the day of anthesis), and expanded fruit (5 days after anthesis) were collected for gene expression analysis. For drought and NaCl stresses, cucumber seedlings at the three-leaf stage with a similar size and height were cultured into 40 L (113 cm × 73 cm × 5 cm) hydroponic pots. The control group was cultured in Hoagland nutrient solution. Drought stress was induced using a Hoagland nutrient solution containing 10% PEG-6000, while NaCl stress was induced using a Hoagland nutrient solution containing 150 mmol/L NaCl (Lu et al., 2022). Leaves were collected at 0, 3, 6, 12, and 24 h after treatment for gene expression analysis. For plant phytohormone treatments, the cucumber seedlings at the three-leaf stage were sprayed with 100 µmol L^-1^ ABA, 100 µmol L^-1^ salicylic acid (SA), 100 µmol L^-1^ jasmonic acid (JA), 50 µmol L^-1^ ethephon (ETH), 50 µmol L^-1^ 2,4-dichlorophenoxyacetic acid (2,4-D), and 50 µmol L^-1^ GA, respectively, while the control condition was sprayed with double distilled water (Li et al., 2019). Plant phytohormone treatments were conducted once a day. After three consecutive days of treatment, cucumber leaves were collected for gene expression analysis. All treatments were performed with three biological replicates.

### 2.4 qRT-PCR analysis

RNA was isolated from various cucumber tissues using the TaKaRa MiniBEST Plant RNA Extraction Kit (TaKaRa, Dalian, China). Isolated RNA was stored at -80°C until further use to prevent degradation. The Mir-X miRNA First-Strand Synthesis Kit (TaKaRa, Dalian, China) was then utilized for first-strand complementary DNA (cDNA) synthesis. qRT-PCR was conducted using the TB Green^®^ Premix Ex TaqTM II (Tli RnaseH Plus) (TaKaRa, Dalian, China), using *U6* snRNA as the endogenous control for Csa-miR159s, and *18S* as the endogenous control for *CsMYBs*. Stem-loop of mature Csa-miR159s was used for qRT-PCR. The specific primer sequences utilized in this study were provided in detail in **Supplementary Table S1**. Gene expression levels were calculated using the 2^-ΔΔCt^ method (Livak and Schmittgen, 2001), each expression level was evaluated using three biological replicates.

## 3 Results

### 3.1 Identification of Csa-miR159s

**Figure 1:**
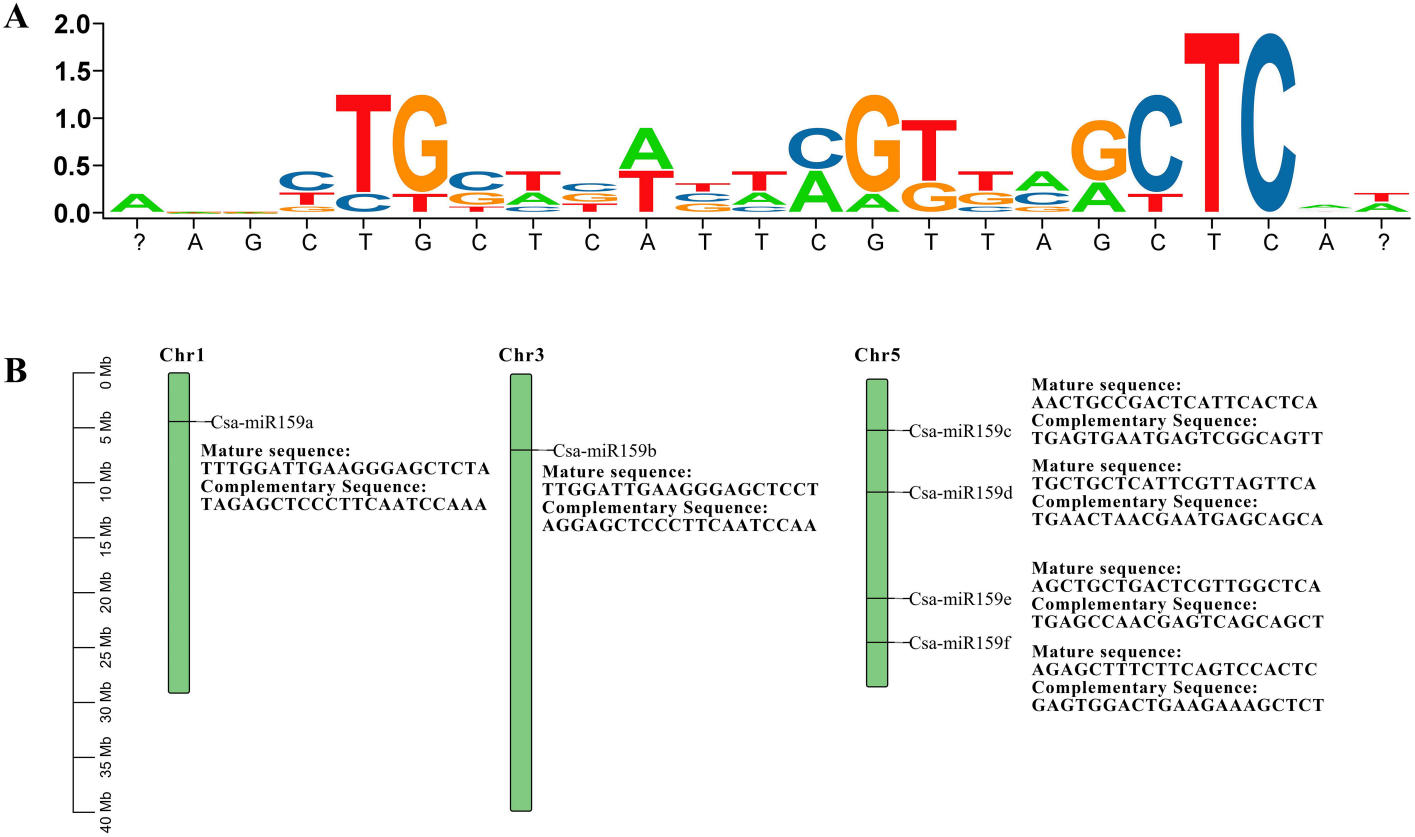
Mature sequences and chromosomal positions of Csa-miR159s. The ratio is measured in megabases (Mb). **(A)** Mature sequences; **(B)** Chromosomal positions.

Six Csa-miR159 sequences were identified from the EnsemblPlants database. The mature sequences of Csa-miR159s were 21-22 nt in length and highly conserved (**Figure 1A**). These sequences were mapped to chromosome 1 (Csa-miR159a), chromosome 3 (Csa-miR159b), and chromosome 5 (Csa-miR159c/d/e/f), respectively, based on their physical positions (**Figure 1B**). Although Csa-miR159c/d/e/f were located on the same chromosome, their mature sequences showed lower similarity. In contrast, Csa-miR159a and Csa-miR159b, which were located on the different chromosomes, shared higher similarity.

**Figure 2:**
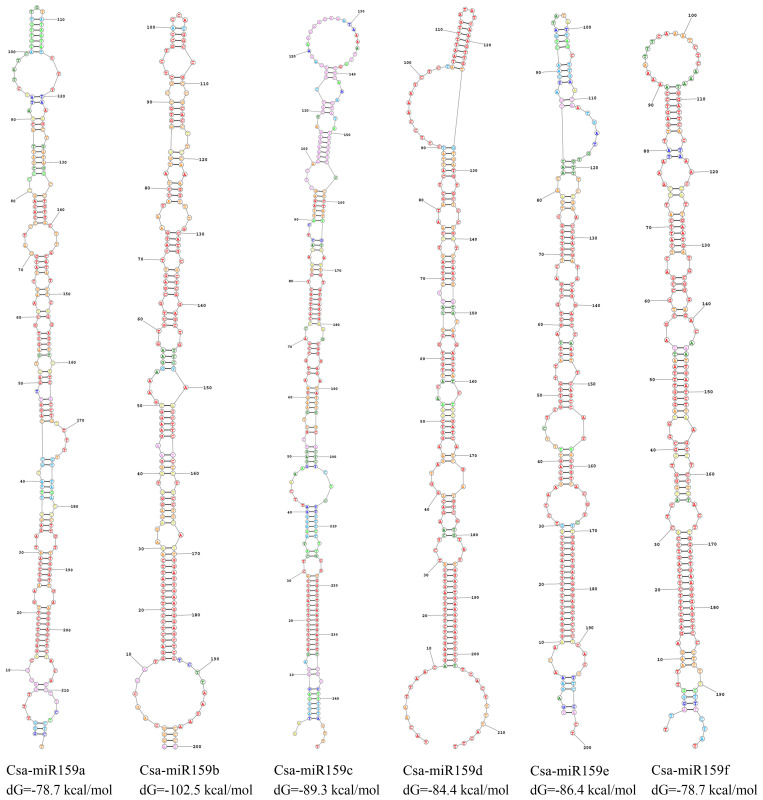
Secondary structures of Csa-miR159s.

The prediction result of secondary structure showed that all pre-miR159s demonstrated a typical stem-loop structures (**Figure 2**). The number of sub-loops varied from 12 (Csa-miR159f) to 18 (Csa-miR159a), and the stem-loop folding free energy ranged from -102.5 kcal/mol (pre-miR159b) to -78.7 kcal/mol (Csa-miR159a/f) (**Figure 2**).

### 3.2 Phylogenetic relationship of miR159s

**Figure 3:**
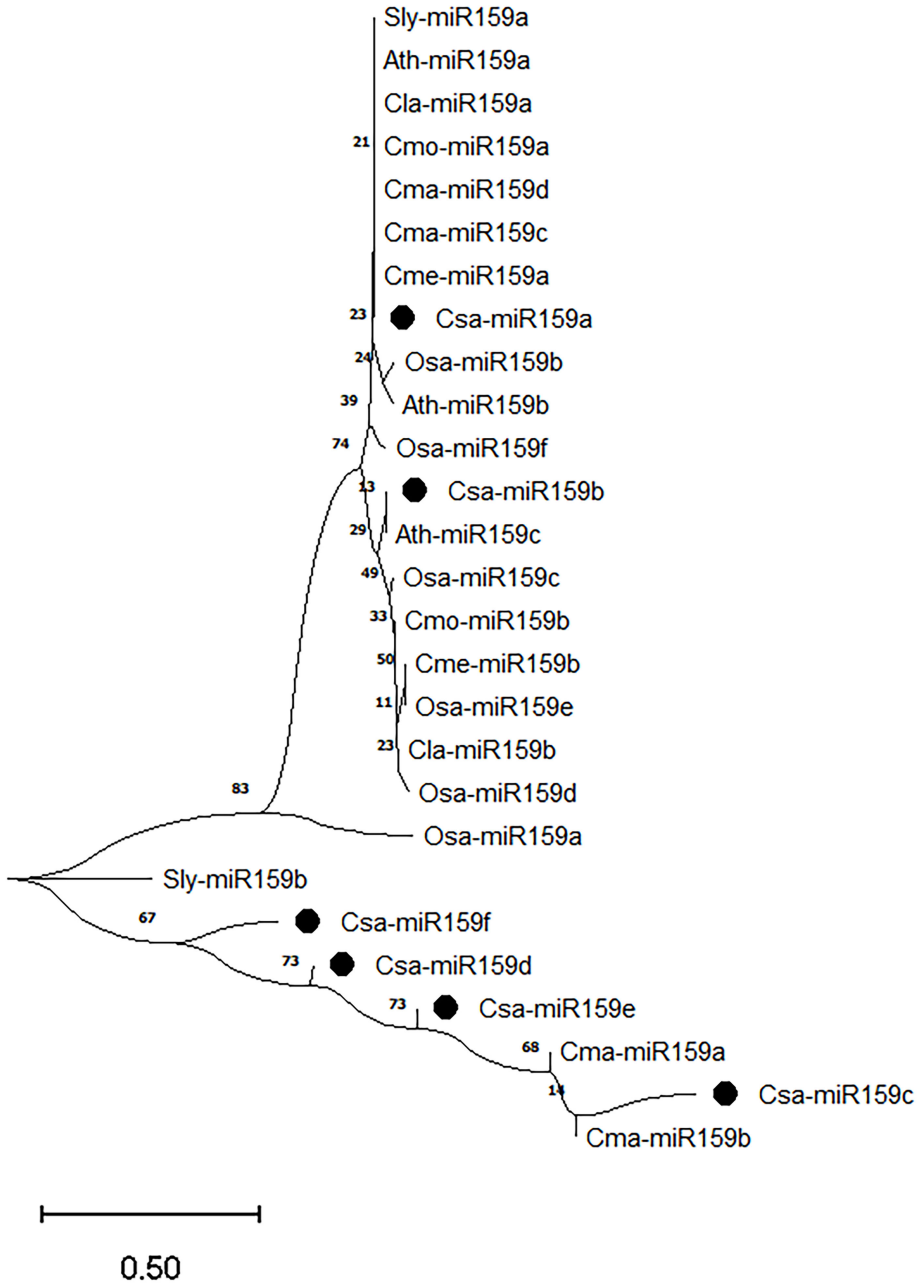
Phylogenetic analysis of miR159s from cucumber, melon, zucchini, pumpkin, watermelon, tomato, rice and *Arabidopsis*. Csa, cucumber; Cme, melon; Cma, zucchini; Cmo, pumpkin; Cla, watermelon; Sly, tomato; Osa, rice; Ath, *Arabidopsis*.

To better understand the evolutionary relationships among miR159s, we further analyzed the mature sequences from cucumber (Csa-miR159a/b/c/d/e/f), melon (Cme-miR159a/b), zucchini (Cma-miR159a/b/c/d), pumpkin (Cmo-miR159a/b), watermelon (Cla-miR159a/b), tomato (Sly-miR159a/b), rice (Osa-miR159a/b/c/d/e/f), and *Arabidopsis* (Ath-miR159a/b/c) (**Figure 3**). Twenty-seven miR159s were classified into two branches based on the evolutionary divergence. Csa-miR159c/d/e/f and Cma-miR159a/b belonged to one branch. Csa-miR159a/b were classified into another branch with remaining members. The phylogenetic tree revealed that cucumber miR159s share closer evolutionary relationships with those of zucchini.

### 3.3 *C*is-regulatory elements analysis of Csa-miR159s

**Figure 4:**
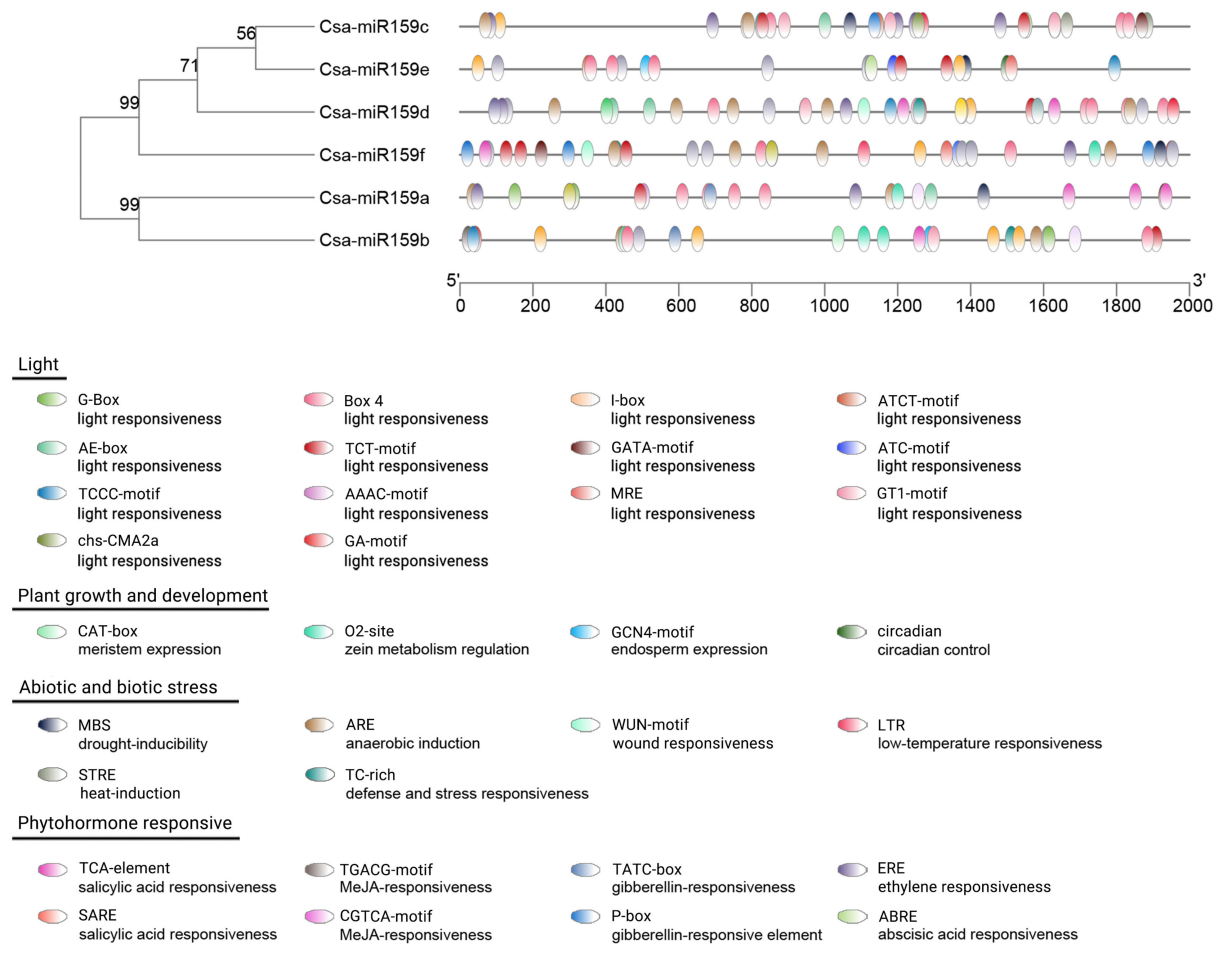
CREs in the promoter regions of Csa-miR159s. Different CRE was presented in the different color shape.

To investigate the potential functions of Csa-miR159s, we analyzed the CREs in the promoter regions. As shown in **Figure 4**, these CREs were grouped into four functional categories. The most abundant category was light response elements, which included Box 4, AAAC-motif, G-Box, TCT-motif, AE-box, GATA-motif, GT1-motif, I-box, GA-motif, TCCC-motif, ATCT-motif, chs-CMA2a, ATC-motif, and MRE. We also detected various phytohormone response elements, such as abscisic acid responsiveness (ABRE), gibberellin responsiveness (TATC-box and P-box), ethylene responsiveness (ERE), MeJA responsiveness (CGTCA-motif, TGACG-motif) and salicylic acid responsiveness (TCA-element, SARE). Furthermore, stress response elements were identified, including anaerobic induction (ARE), drought inducibility (MBS), heat induction (STRE), low temperature responsiveness (LTR), wound responsiveness (WUN-motif), and defense and stress responsiveness (TC-rich). Additionally, regulatory elements related to plant growth and development were also identified, such as zein metabolism regulation (O2-site), meristem expression (CAT-box), endosperm expression (GCN4-motif) and circadian control (circadian).

### 3.4 Prediction of target genes of Csa-miR159s

To investigate the regulatory mechanisms of Csa-miR159s, potential target genes were predicted using the psRNA-Target tool (**Table 1**). All target genes were named according to their subgroups and chromosomal positions from top to bottom (from *Cs1RMYB* to *Cs4RMYB*). The results revealed that *Cs2RMYB37*, *Cs2RMYB64*, *Cs1RMYB31*, *Cs1RMYB9* and *Cs2RMYB25* were recognized as the target genes of Csa-miR159a. Similarly, *Cs2RMYB37*, *Cs2RMYB64*, *Cs1RMYB31*, *Cs1RMYB9* and *Cs3RMYB1* were predicted as the target genes of Csa-miR159b. Additionally, *Cs2RMYB27* and *Cs2RMYB32* were found to be the target genes of Csa-miR159d. However, no target genes were detected for Csa-miR159c, Csa-miR159e and Csa-miR159f. Notably, all predicted target genes were classified as MYB or MYB-like transcription factors.

**Table 1:** Target genes prediction of Csa-miR159s.

miRNA_ACC	Rename	Target_Acc	Expectation	Target regions	Inhibition	Multiplicity	Description
Csa-miR159a	*Cs2RMYB37*	Csa4G022940.1	0.5	CDS:2507239-2508535	Cleavage	1	MYB-related transcription factor
	*Cs2RMYB64*	Csa7G043580.1	0.5	CDS:2401528-2403473	Cleavage	1	MYB transcription factor
	*Cs1RMYB31*	Csa6G105150.1	2.0	CDS:6908538-6908833	Cleavage	1	MYB-like transcription factor
	*Cs1RMYB9*	Csa2G035350.1	3.0	CDS:3526253-3527734	Translation	1	MYB transcription factor
	*Cs2RMYB25*	Csa3G264750.1	5.0	CDS:16264809-16266219	Cleavage	1	MYB family transcription factor
Csa-miR159b	*Cs2RMYB37*	Csa4G022940.1	0.5	CDS:2507239-2508535	Cleavage	11	MYB-related transcription factor
	*Cs2RMYB64*	Csa7G043580.1	0.5	CDS:2401528-2403473	Cleavage	1	MYB transcription factor
	*Cs1RMYB31*	Csa6G105150.1	2.0	CDS:6908538-6908833	Cleavage	1	MYB-like transcription factor
	*Cs1RMYB9*	Csa2G035350.1	3.0	CDS:3526253-3527734	Translation	1	MYB transcription factor
	*Cs3RMYB1*	Csa2G375240.1	5.0	CDS:18863762-18872002	Cleavage	1	Putative MYB transcription factor
Csa-miR159d	*Cs2RMYB27*	Csa3G386830.1	4.5	CDS:18944049-18946409	Cleavage	1	Putative MYB transcription factor
	*Cs2RMYB32*	Csa3G816030.1	5.0	CDS:31548636-31550625	Cleavage	1	MYB transcription factor

### 3.5 Expression profiles of Csa-miR159s and their target genes

**Figure 5:**
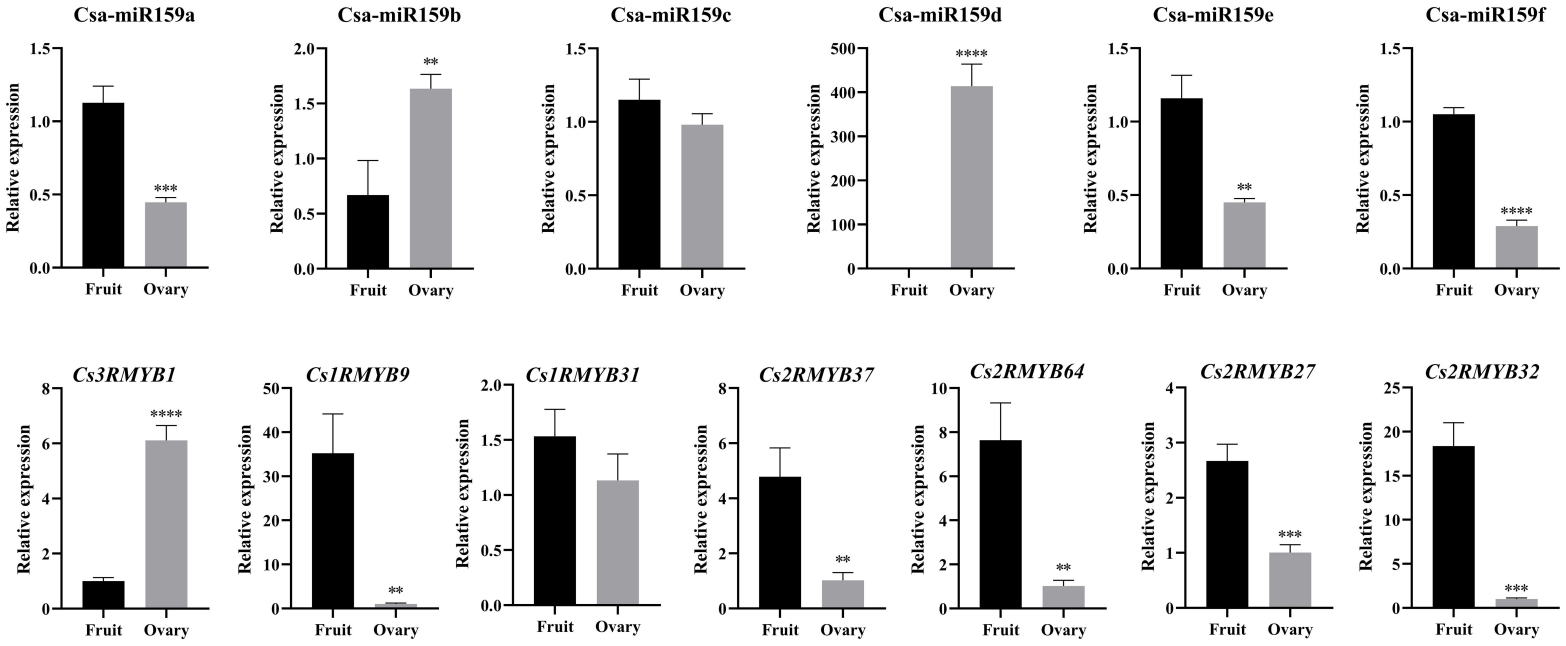
Relative expression of Csa-miR159s and *CsMYBs* in the ovary and the expanded fruit of cucumber by qRT-PCR. The X-axis indicated the tested tissue samples. Error bars represented ± standard deviation (SD) with three biological replicates. Different asterisks above the bars indicated significant differences. (** *p* < 0.01, *** *p* < 0.001, **** *p* < 0.0001).

In this study, qRT-PCR was used to confirm the expression profiles of Csa-miR159s and their target genes in the ovary and expanded fruit (**Figure 5**). The expression of Csa-miR159b in the ovary was 2.44-fold higher than that in the expanded fruit, while the expression levels of Csa-miR159a/c/e/f in the ovary were lower than those in the expanded fruit. Notably, Csa-miR159d was only expressed in the ovary and was not detected in the expanded fruit. In terms of the target genes, *Cs1RMYB9*, *Cs1RMYB31*, *Cs2RMYB37*, *Cs2RMYB64*, *Cs2RMYB27* and *Cs2RMYB32* showed lower expression in the ovary compared to the expanded fruit, except for *Cs3RMYB1* which exhibited the opposite trend. These findings suggested that Csa-miR159b might negatively regulate cucumber fruit expansion by targeting *Cs1RMYB9*, *Cs1RMYB31*, *Cs2RMYB37* and *Cs2RMYB64*. Additionally, Csa-miR159d might negatively regulate cucumber fruit expansion by targeting *Cs2RMYB27* and *Cs2RMYB32*. Taken together, these results highlighted the tissue specificity and functional diversity of Csa-miR159s.

### 3.6 Csa-miR159s response to abiotic stresses and plant phytohormones

**Figure 6:**
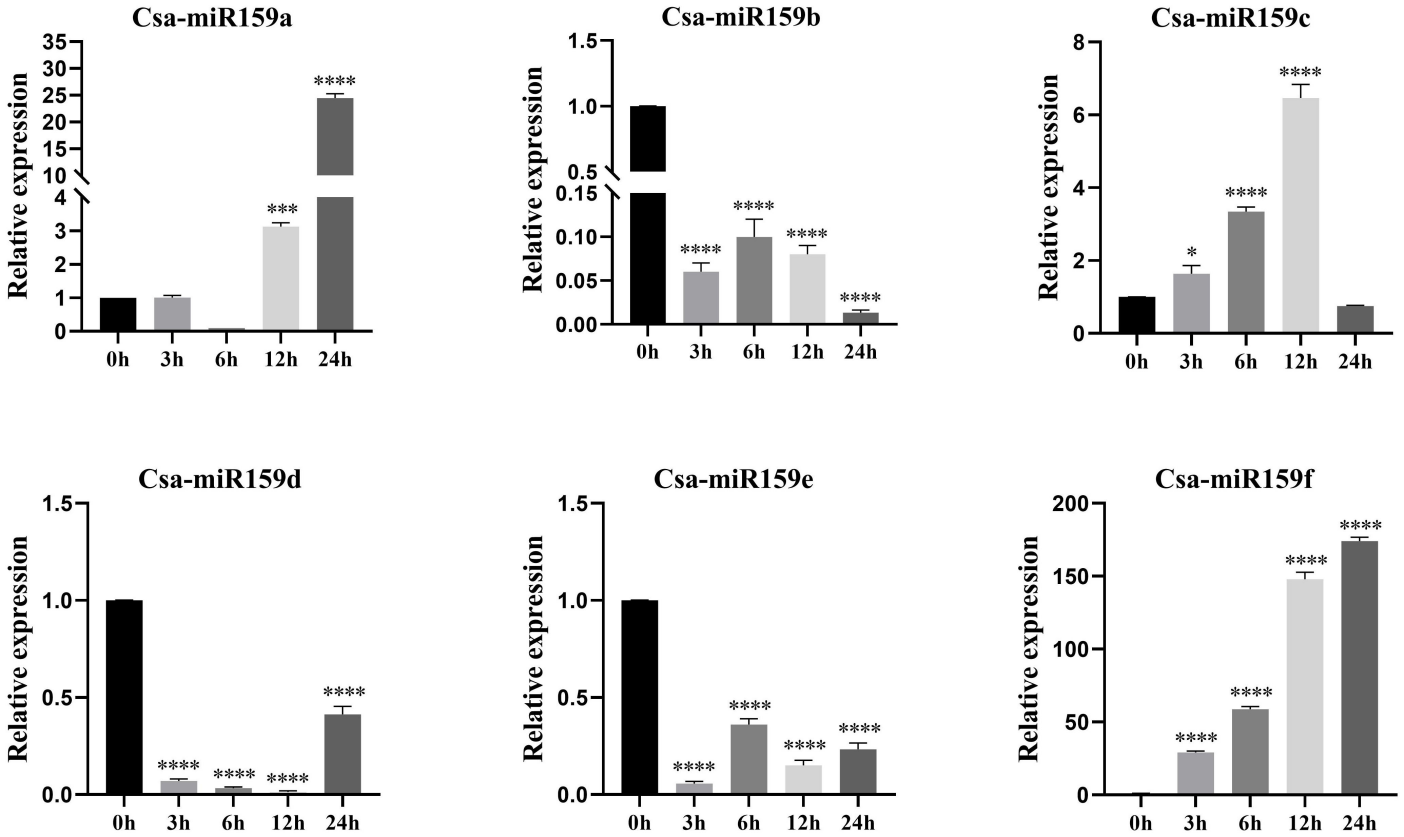
Relative expression of Csa-miR159s in response to 10% PEG-6000 treatment (0, 3, 6, 12 and 24 h). Error bars represented ± standard deviation (SD) with three biological replicates. Different asterisks above the bars indicated significant differences. (* *p* < 0.05, *** *p* < 0.001, **** *p* < 0.0001).

**Figure 7:**
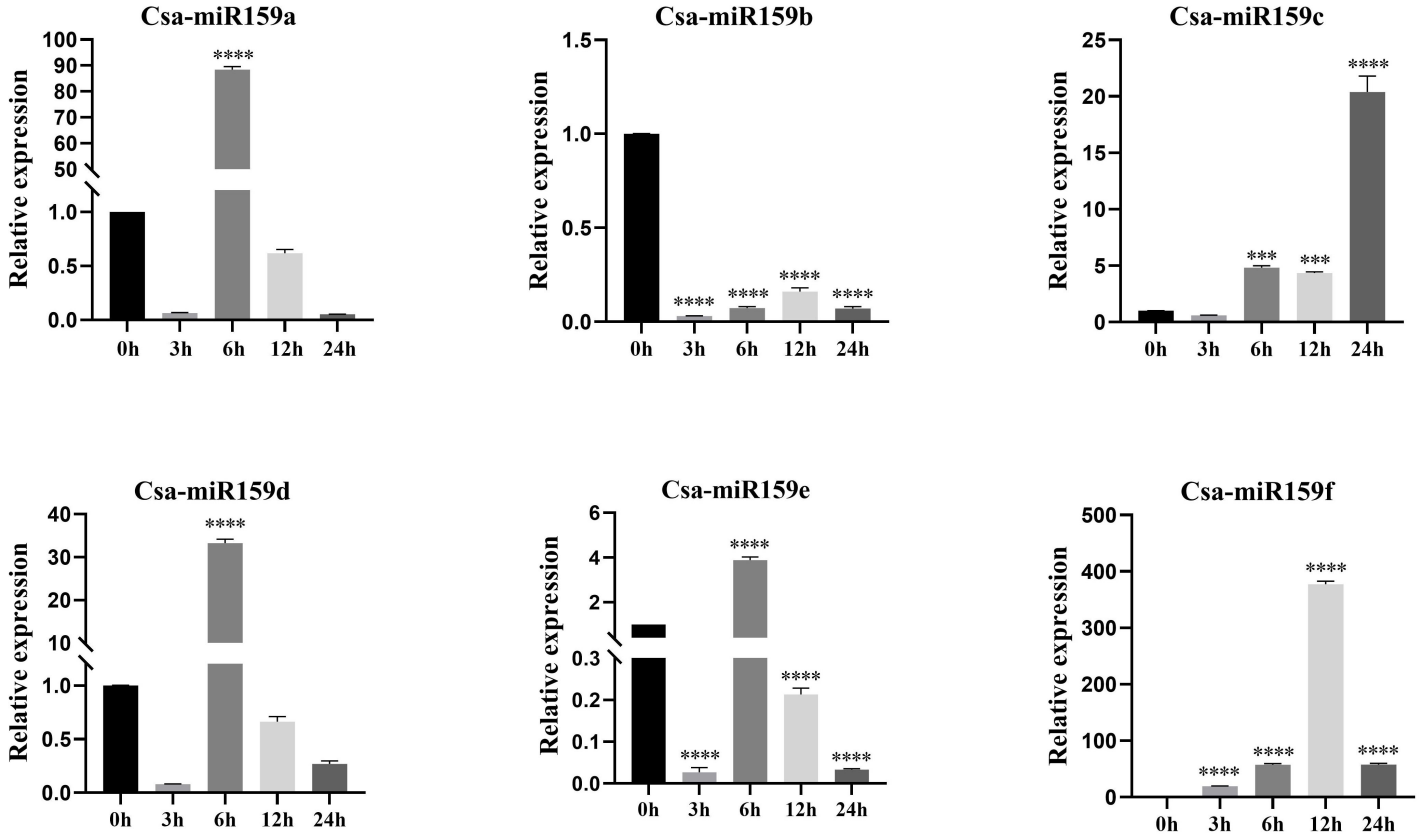
Relative expression of Csa-miR159s in response to 150 mmol/L NaCl treatment (0, 3, 6, 12 and 24 h). Error bars represented ± standard deviation (SD) with three biological replicates. Different asterisks above the bars indicated significant differences. (*** *p* < 0.001, **** *p* < 0.0001).

**Figure 8:**
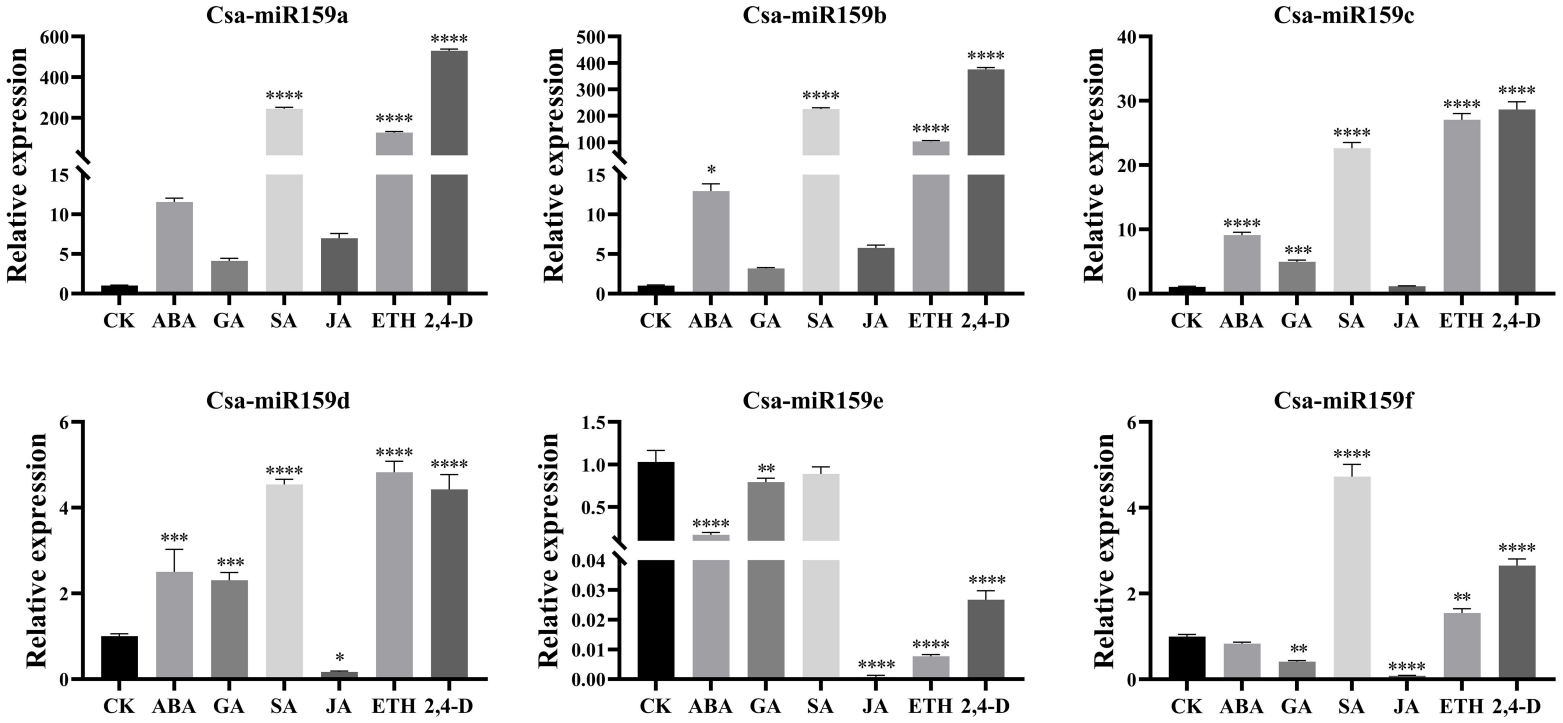
Relative expression of Csa-miR159s in response to different plant phytohormones, such as, ABA, GA, SA, JA, ETH and 2,4-D. Error bars represented ± standard deviation (SD) with three biological replicates. Different asterisks above the bars indicated significant differences. (* *p* < 0.05, ** *p* < 0.01, *** *p* < 0.001, **** *p* < 0.0001).

The study investigated the expression patterns of Csa-miR159s under different treatments including PEG, NaCl and plant phytohormones. Under PEG stress, the expression levels of Csa-miR159a/f increased gradually, reaching the top at 24 h after treatment. Specifically, Csa-miR159a was 24.4-fold higher than the control, and Csa-miR159f was 174.1-fold higher. Conversely, Csa-miR159c exhibited the increased expression, peaking at 12 h, and then decreasing to the lowest level at 24 h after treatment, 1.3-fold lower than the control. However, Csa-miR159b/d/e were significantly down-regulated under PEG stress. Compared to the control, their expression levels decreased by 76.9, 2.4 and 4.3-fold, respectively (**Figure 6**). Under NaCl stress, Csa-miR159a/d/e were all significantly up-regulated at 6 h, with increases of 88.5, 33.2 and 3.9-fold compared to the control. While Csa-miR159b was remarkably down-regulated from 3 h to 24 h. In contrast, the expression levels of Csa-miR159c initially decreased at 3 h, then increased at 6 h, and peaked at 24 h. Csa-miR159f showed the increased expression, peaking at 12 h, and then decreasing at 24 h (**Figure 7**). In relation to plant phytohormones, it was observed that Csa-miR159a/b/c were significantly induced by SA, ETH and 2,4-D. On the other hand, Csa-miR159d showed significant up-regulation in response to ABA, GA, SA, ETH, and 2,4-D. Furthermore, Csa-miR159e exhibited a remarkable down-regulation when exposed to ABA, GA, JA, ETH, and 2,4-D. Additionally, Csa-miR159f displayed significant up-regulation specifically in response to SA, ETH, and 2,4-D (**Figure 8**). These findings indicated that Csa-miR159s might be involved in plant stress response and phytohormone regulation.

## 4 Discussion

miRNA plays a crucial role in plant growth and development, and stress response by regulating the expression of their target genes. Among these miRNAs, miR159 has been extensively identified and characterized in numerous plant species, such as *Arabidopsis* (Palatnik et al., 2007), grape (Zhang et al., 2019), soybean (Li et al., 2023) and *Dendrobium officinale* (Hao and Zhang, 2022). However, there is limited research on the functions of Csa-miR159s regarding fruit expansion and abiotic stress response in cucumber. In this study, we identified six Csa-miR159s and their target genes. Csa-miR159s were unevenly distributed on chromosome 1, chromosome 3, and chromosome 5. Interestingly, Csa-miR159c/d/e/f, which had different mature sequences, were located on the same chromosome. Conversely, Csa-miR159a/b, which had higher sequence homology, were distributed on the different chromosomes. This suggested that the divergent chromosomal localization of Csa-miR159s could be a result of gene duplication events or evolutionary pressures that had caused their dispersion across different chromosomes, and this dispersion might have promoted the diversified functions of Csa-miR159s. Phylogenetic analysis revealed a close relationship between miR159s in cucumber and those in zucchini, possibly due to common evolutionary processes as the members of the *Cucurbitaceae* family.

The promoter region typically contains specific CREs with distinct functions. Analysis of CREs can offer insights into the potential functions of genes in the growth and development, and stress response. In our study, we identified stress-related elements in Csa-miR159s, including MBS, ARE, LTR, STRE and TC-rich, which suggested that Csa-miR159s might be associated with stress response in cucumber. Previous studies have demonstrated that up-regulated expression of miR159 enhanced stress tolerance in *Arabidopsis* (Reyes and Chua, 2007; Liu et al., 2008) and sweet potato (Yang et al., 2020). Conversely, some reports indicated that miR159 was down-regulated under salt stress and drought stress (Yang et al., 2020), and overexpression of miR159 increased stress sensitivity in rice (Wang et al., 2012) and potato (Pieczynski et al., 2013). In addition, Peng et al. (2018) observed that the expression of miR159 in rice was down-regulated after 3 h of salt stress treatment, followed by up-regulation. Our results confirmed that Csa-miR159s could respond to PEG and NaCl stresses by qRT-PCR, and significant differences were observed in their expression patterns. Additional experimental validation is necessary to elucidate the transcriptional regulation mechanisms of Csa-miR159s under PEG and NaCl stresses.

Several studies have indicated that miR159 can respond to some plant phytohormones, including ABA (Reyes and Chua, 2007) and GA (Wang et al., 2017). For instance, the application of exogenous GA led to a significant decrease in the expression level of Fa-miR159a, while the expression level of Fa-miR159b remained unchanged in strawberry (Csukasi et al., 2012). Similarly, exogenous GA treatment resulted in the up-regulated expression of VvmiR159c during flowering, whereas VvmiR159a/b showed no significant changes in grape (Wang et al., 2018). In this study, CREs of six Csa-miR159s included various plant phytohormone response elements such as ABA, GA, SARE, ABRE, MeJA and ETH. Csa-miR159s exhibited distinct expression patterns under ABA, GA, SA, JA, ETH and 2,4-D treatments. This suggested that Csa-miR159s might play a significant role in plant phytohormone signaling pathways with varying response mechanisms.

Fruit development is a crucial stage in the life cycle of plants, encompassing a variety of intricate physiological and molecular processes. miR159 has been found to be crucial for ovule development and fruit set in tomato. Overexpression of Sly-miR159 caused abnormal ovule development, premature maturation, and seedless fruit in tomato (da Silva et al., 2017; Deng, 2020). Furthermore, Sly-miR159-*SlGAMYB2* pathway has been identified to regulate fruit morphology, whereby inhibition of Sly-miR159 led to larger fruit and a reduced length/width ratio (Zhao et al., 2022). Here, we discovered that the expression of Csa-miR159b/d was significantly higher in the ovary than that in the expanded fruit. Conversely, the expression levels of Csa-miR159a/c/e/f in the ovary were lower than those in the expanded fruit. These findings suggested that Csa-miR159s could be involved in fruit expansion in cucumber. miRNA regulates the growth and development in plants by inhibiting the expression of its target genes. In this study, *Cs2RMYB37*, *Cs2RMYB64*, *Cs1RMYB31*, *Cs1RMYB9* and *Cs3RMYB1* were predicted as the target genes of Csa-miR159b based on the PsRNA-Target results. While, *Cs2RMYB27* and *Cs2RMYB32* were found to be the target genes of Csa-miR159d. Furthermore, we found that *Cs1RMYB9*, *Cs1RMYB31*, *Cs2RMYB37*, *Cs2RMYB64*, *Cs2RMYB27* and *Cs2RMYB32* showed lower expression in the ovary compared to the expanded fruit by qRT-PCR. These findings suggested that Csa-miR159b might negatively regulate cucumber fruit expansion by targeting *Cs1RMYB9*, *Cs1RMYB31*, *Cs2RMYB37* and *Cs2RMYB64*. Similarly, Csa-miR159d might negatively regulate cucumber fruit expansion by targeting *Cs2RMYB27* and *Cs2RMYB32*. Taken together, our results suggested a vital role of Csa-miR159s in fruit expansion and stress response in cucumber. Further research is required to comprehend the functions of Csa-miR159s by performing a gain of function or loss of function assay.

## 5 Conclusions

In this study, six miR159 family members were identified in cucumber. Bioinformatics and expression profiles of Csa-miR159s were performed to discover their potential functions. The results showed that Csa-miR159s played a crucial role in the response to PEG, NaCl and plant phytohormones. Additionally, it was found that Csa-miR159b/d might inhibit the cucumber fruit expansion by targeting their target genes. Our study provided a theoretical foundation for further investigation into the roles of Csa-miR159s under fruit expansion and abiotic stresses in cucumber.

## Data Availability

The original contributions presented in the study are included in the article/**Supplementary Material**. Further inquiries can be directed to the corresponding author.
