# Supplementary material for: Identification and characterization of Csa-miR159s and their expression patterns under different abiotic stresses in cucumber (Cucumis sativus L.)
Source: Front Plant Sci. 2025 Apr 24;16:1518406. doi: 10.3389/fpls.2025.1518406 (PMC12058866; doi:10.3389/fpls.2025.1518406)
Supplement: Supplementary file 1 [file Table1.docx]

Primer Sequences for Csa-miR159s and *CsMYBs*

| Primer Name | Primer Sequence（5’–3’） |
| --- | --- |
| *U6* snRNA-F | GGAGGCAGAGGCATTGGA |
| *U6* snRNA-R | CCCACCTTTGTTACCACCTTTG |
| Csa-miR159a-F | TGCTTTGGATTGAAGGGAGCT |
| Csa-miR159b-F | CGGTTGGATTGAAGGGAGC |
| Csa-miR159c-F | CGGCAACTGCCGACTCATT |
| Csa-miR159d-F | GCCGTGCTGCTCATTCGTT |
| Csa-miR159e-F | TGCTGACTCGTTGGCTCAA |
| Csa-miR159f-F | CGGCAGAGCTTTCTTCAGT |
| Csa-miR159a/b/c/d/e/f-R | CAGTGCAGGGTCCGAGGTAT |
| *18S*-F | CAACCATAAACGATGCCGA |
| *18S*-R | AGCCTTGCGACCATACTCC |
| *Cs2RMYB37*-F | AATGCCTGTCTTAGAGGTTCCTAGC |
| *Cs2RMYB37*-R | ACTGTACTGATAGCGTCGCCATC |
| *Cs2RMYB64*-F | TTGCCGCTTTACCCTCTTGAAATTC |
| *Cs2RMYB64*-R | TTATGGTGGTGATGGTGGTGGTG |
| *Cs1RMYB31*-F | AGGGCCATGGAAACTCGAAGAAG |
| *Cs1RMYB31*-R | TTGCCAGTCCGCTGAAGAAGG |
| *Cs1RMYB9*-F | TGGCTTCTTGTGGCACTCAGAC |
| *Cs1RMYB9*-R | TGTGGCGGTGATTGATTTGATGATG |
| *Cs3RMYB1*-F | TCTACACCAATGAATCCACCGACTC |
| *Cs3RMYB1*-R | ATCCTTTGAACCCTGCTGCTTTTG |
| *Cs2RMYB27*-F | CGGCGGAGAATGGTGAGTCG |
| *Cs2RMYB27*-R | ACCTTCAAATCCTTGTTGGTTTCCC |
| *Cs2RMYB32*-F | CGGAGAACGATGACGCTACGG |
| *Cs2RMYB32*-R | CGCCTTCTTCAAACAGCCACTTC |
